# Nisin J, a Novel Natural Nisin Variant, Is Produced by Staphylococcus capitis Sourced from the Human Skin Microbiota

**DOI:** 10.1128/JB.00639-19

**Published:** 2020-01-15

**Authors:** Julie N. O’Sullivan, Paula M. O’Connor, Mary C. Rea, Orla O’Sullivan, Calum J. Walsh, Brian Healy, Harsh Mathur, Des Field, Colin Hill, R. Paul Ross

**Affiliations:** a Teagasc Food Research Centre, Fermoy, County Cork, Ireland; b School of Microbiology, University College Cork, Cork, Ireland; c APC Microbiome Ireland, University College Cork, Cork, Ireland; Geisel School of Medicine at Dartmouth

**Keywords:** antimicrobial agents, antimicrobial peptides, antimicrobial structure, bacteriocins, microbiota, natural antimicrobial products, skin microbiota, nisin

## Abstract

The skin microbiota is thought to play a key role in host protection from infection. Nisin J is a novel nisin variant produced by Staphylococcus capitis APC 2923, a strain isolated from the toe web space area in a screening study performed on the human skin microbiota. Whole-genome sequencing and mass spectrometry of the purified peptide confirmed that *S. capitis* APC 2923 produces a 3,458-Da bacteriocin, designated nisin J, which exhibited antimicrobial activity against a range of Gram-positive pathogens, including methicillin-resistant Staphylococcus aureus (MRSA) and Cutibacterium acnes. The gene order in the nisin J gene cluster (*nsjFEGBTCJP*) differs from that of other nisin variants in that it is lacking the nisin regulatory genes, *nisRK*, as well as the nisin immunity gene *nisI*. Nisin J has 9 amino acid changes compared to prototypical nisin A, with 8 amino acid substitutions, 6 of which are not present in other nisin variants (Ile4Lys, Met17Gln, Gly18Thr, Asn20Phe, Met21Ala, Ile30Gly, Val33His, and Lys34Thr), and an extra amino acid close to the C terminus, rendering nisin J the only nisin variant to contain 35 amino acids. This is the first report of a nisin variant produced by a *Staphylococcus* species and the first nisin producer isolated from human skin.

**IMPORTANCE** This study describes the characterization of nisin J, the first example of a natural nisin variant, produced by a human skin isolate of staphylococcal origin. Nisin J displays inhibitory activity against a wide range of bacterial targets, including MRSA. This work demonstrates the potential of human commensals as a source for novel antimicrobials that could form part of the solution to antibiotic resistance across a broad range of bacterial pathogens.

## Abstract (precis)

This study describes the characterization of nisin J, the first example of a natural nisin variant, produced by a human skin isolate of staphylococcal origin. Nisin J displays inhibitory activity against a wide range of bacterial targets, including MRSA. This work demonstrates the potential of human commensals as a source for novel antimicrobials that could form part of the solution to antibiotic resistance across a broad range of bacterial pathogens.

## INTRODUCTION

The human skin microbiome is home to ∼10^12^ bacteria (1), and interest in the potential of skin bacteria to produce antimicrobials is growing, given the spread of antibiotic resistance (AR). Staphylococcus capitis is a member of the resident skin microbiota. First isolated from human skin in 1975, it has since been regarded as an opportunistic pathogen and has been associated with sepsis in neonates, meningitis, and endocarditis (2). Little is known about the inhibitory nature or antimicrobial activity of *S. capitis*, with only one report of *S. capitis* EPK-1 producing the glycylglycine endopeptidase ALE-1, an enzyme that targets the cell wall of Staphylococcus aureus (3). More recently, genomic analysis of an *S. capitis* strain isolated from the skin of a human toe revealed the presence of gene clusters capable of encoding gallidermin, epidermin, and phenol soluble modulins, highlighting its potential to produce antimicrobial peptides (AMPs) (4). In a recent study, our group detected antimicrobial activity by a number of *S. capitis* strains isolated from different areas of the human skin (5) and highlighted the potential for *S. capitis* species to produce bacteriocins (small ribosomally synthesized peptides produced by a range of bacteria which kill other bacteria). Interestingly, bacteriocin production is considered to be a probiotic trait in that bacteriocins function in helping the producer strain to become established in a niche, by killing off competitors and interacting with the immune system. Although the impact of nisin on immune systems has not yet been completely elucidated, this peptide stimulates a wide array of effects, and it influences various populations of cells involved in immunity (6–12).

One of the oldest known and most intensively studied bacteriocins is nisin, which was first described in this journal by Rogers and Whittier in 1928 (13). Nisin has been used in food preservation since 1953 (14) and was granted generally regarded as safe (GRAS) status in 1988 by the Food and Drug Administration (FDA). It is also approved by the World Health Organization (WHO) as a food additive and has been assigned the E number E234. Since the discovery of nisin, interest in bacteriocins has grown rapidly. Nisin A, composed of 34 amino acids, is produced by several strains of Lactococcus lactis (15). Nisin is a lantibiotic and thus a member of the class I bacteriocins (16). Lantibiotics are small peptides (<5 kDa) and are produced by many Gram-positive bacteria to inhibit or kill other Gram-positive bacteria (17). Production of other lantibiotics is common among commensal coagulase-negative staphylococci. For example, Staphylococcus gallinarum, Staphylococcus epidermidis, and Staphylococcus hominis produce the lantibiotics gallidermin, epidermin, and hominicin, respectively (18–20). Class I bacteriocins consist of posttranslationally modified bacteriocins which are subdivided into 4 classes, as follows: class Ia, lanthipeptides (of which nisin is the most prominent member); class Ib, head-to-tail cyclized peptides; class Ic, sactibiotics; and class Id, linear azol(in)e-containing peptides (8, 21). Lantibiotics are characterized by the presence of lanthionine/β-methyllanthionine residues and are produced through the dehydration of serine and threonine residues to form dehydroalanines and dehydrobutyrines, respectively. These dehydrated residues in turn react with cysteine thiols, forming lanthionine bridges (22, 23). The lantibiotics are subdivided based on the enzymes catalyzing the formation of lanthionines. Subclass I requires two distinct enzymes, LanB and LanC, whereas subclass II is modified by a single enzyme, LanM. Subclass III has no associated antimicrobial activity and is modified by a single enzyme, LanKC, while subclass IV is modified by LanL (24). Studies have revealed that nisin and other structurally related lantibiotics use the membrane-bound peptidoglycan precursor lipid II as a docking molecule, which consequently promotes two bactericidal activities, pore formation and inhibition of peptidoglycan biosynthesis (25). Significantly, lantibiotics have been shown to possess activity against antibiotic-resistant targets such as vancomycin-resistant enterococci (VRE) and methicillin-resistant Staphylococcus aureus (MRSA) and may have the potential to mitigate the looming global AR crisis (26).

**FIG 1:**
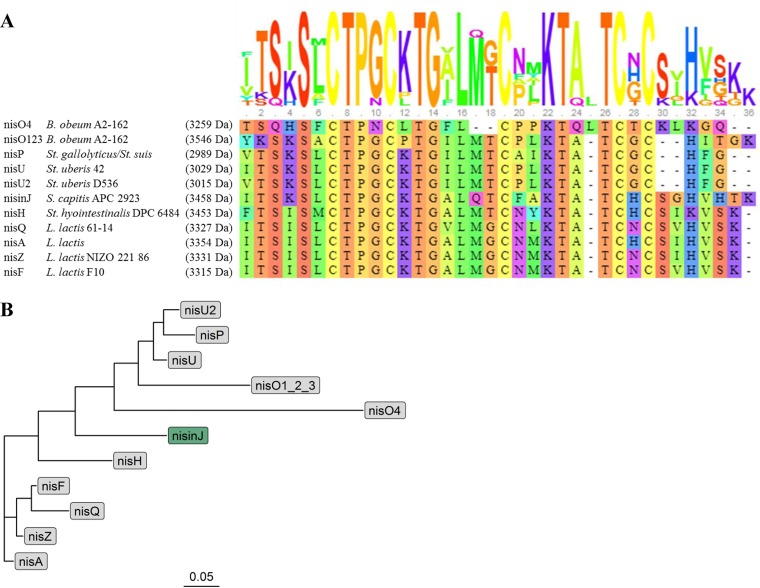
(A) Visualization of the multiple-sequence alignment from MUSCLE (plotted using http://msa.biojs.net/app/) of all natural nisin (nis) variants aligned with strain origin. The total height of the sequence logo at each position reflects the degree of conservation at that position in the alignment, while the height of each letter in that position is proportional to the observed frequency of the corresponding amino acid at that position. Nisin A (13), nisin Z (48), nisin F (49), nisin Q (50), nisin H (27), nisin J (5), nisins U and U_2_ (51), nisin P (52, 53), and nisins O_1_ to O_4_ (54) are shown. *L*., *Lactococcus*; *S*., *Staphylococcus*; *B*., *Blautia*; *St*., *Streptococcus*. (B) Dendrogram showing phylogenetic relatedness in primary structures of all known natural nisin variants, suggesting the possible existence of an evolutionary link between the nisin-producing species. The order in which they branch shows the relatedness between them, and the branch length represents phylogenetic distance (0.05 represents a scale for the phylogenetic distance).

A number of nisin variants have been discovered since the original nisin A was characterized (Fig. 1A). Nisin variants of lactococcal origin are more similar to each other than to variants from other genera such as *Streptococcus* (Fig. 1B). Nisin Z is the most closely related nisin variant to nisin A, with only a single amino acid substitution, His27Asn. Nisin U, U_2_, and P each contain 31 amino acids, nisins O_1–3_ contain 33 amino acids, and nisin O_4_ contains 32 amino acids, making them shorter than other previously described nisin variants. Here, we describe nisin J, produced by the *S. capitis* strain APC 2923, isolated in a screening study of the human skin microbiota. At 35 amino acids, nisin J is the longest nisin variant identified to date and has antimicrobial activity against significant human pathogens, including staphylococci, streptococci, and Cutibacterium acnes.

## RESULTS

### A nisin-like gene cluster exists within the *S. capitis* APC 2923 genome.

**FIG 2:**
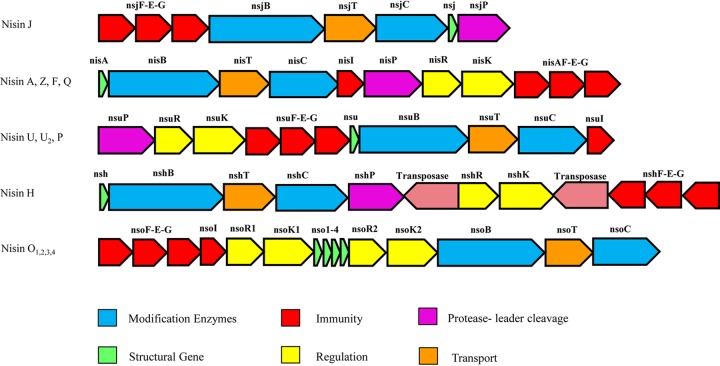
Comparison of bacteriocin gene clusters of different nisin variants.

*S. capitis* APC 2923 was previously isolated from the toe web space area in a screening study of the human skin microbiota that sought to identify novel antimicrobial-producing strains (5). This strain was of particular interest due to its potent activity against the indicator strain Lactobacillus delbrueckii subsp. bulgaricus LMG 6901 and its broad inhibitory spectrum against a panel of Staphylococcus, Streptococcus, and Corynebacterium species and against Cutibacterium acnes. Whole-genome sequencing of this strain revealed a nisin gene cluster of ∼9.78 kb compared to ∼13.3 kb for nisin A. The structural gene *nisJ* encodes a peptide with the following eight amino acid variations compared to nisin A: Ile4Lys, Met17Gln, Gly18Thr, Asn20Phe, Met21Ala, Ile30Gly, Val33His, and Lys34Thr. Nisin J also contains an extra amino acid at the C terminus, making nisin J the longest nisin variant identified to date (Fig. 1A). A dendrogram of the natural nisin variants (Fig. 1B) demonstrates that peptides which have a closer common ancestor are more similar than are peptides than have more distant branching points. Lactococcal nisin variants are structurally distinct from all other nisin variants. Staphylococcal nisin J groups in the middle of the tree and appears to be more similar to streptococcal nisin than to lactococcal nisins. Nisins of Blautia origin appear to be more phylogenetically distinct due to longer branching. Streptococcal nisins H and J are more closely related to lactococcal nisins than to other streptococcal nisins, U, U_2_, and P. The gene order of the nisin J cluster (*FEGBTCJP*) also differs from that of the nisin A in that it contains eight as opposed to the 11 genes within the cluster (Fig. 2). The BAGEL4 bacteriocin genome mining tool predicted that the nisin J prepeptide is composed of 61 amino acids with a leader sequence consisting of 26 amino acids. Overall, the nisin J mature peptide has 62.5% identity to the nisin H structural peptide produced by Streptococcus hyointestinalis (27). The identity and function of features of the nisin J operon are listed in Table 1.

**TABLE 1:** Identity and function of features of the draft pJOS01 plasmid sequence^*a*^

Feature name	Position of:		Size (aa)	E value	Putative function (conserved domain)	% identity to best match
	Start codon	Stop codon				
J1	3	518	172	3E−124	DDE_Tnp_IS*240* superfamily; Rve transposase	100 to IS*6* family transposase of S. epidermidis
J2	562	1221	220	6E−149	ABC2_membrane superfamily; NosY ABC-type transport system involved in multicopper enzyme maturation, permease component	100 to ABC transporter permease subunit of *S. capitis*
J3	1749	1874	125	3E−17	DUF2648 superfamily; unknown function	100 to multiple species; DUF2648 domain-containing protein
J4	1886	3385	500	0	NADB_Rossmann superfamily; MqO malate:quinone oxidoreductase	100 to multiple species; malate dehydrogenase (quinone) (*Staphylococcus*)
J5	3446	5050	535	0	l-Lactate permease superfamily (energy production and conversion)	100 to *L. lactate* permease (*Staphylococcus*)
J6	5085	5789	235	6E−171	Alpha-acetolactate decarboxylase superfamily (secondary metabolite biosynthesis, transport, and catabolism)	100 to alpha-acetolactate decarboxylase
J7	5823	7487	555	0	Acetolactate synthase superfamily (PRK08617) (amino acid transport and metabolism, coenzyme transport, and metabolism)	100 to acetolactate synthase
J8	8213	8413	67	8E−39	CspA family (transcription) DNA binding domain	100 to cold shock protein (*Staphylococcus*)
CdR	8807	9424	206	1E−139	Cadmium resistance transporter superfamily; CadD protein, predicted permease (inorganic ion transport and metabolism)	100 to cadmium resistance transporter (Mycobacteroides abscessus subsp. *massiliense*)
J10	9442	9789	116	4E−74	Arsenical resistance operon repressor family; DNA-binding transcriptional regulator (transcription)	100 to HTH transcriptional regulator (*Staphylococcus*)
J11	10002	10610	203	2E−144	Serine recombinase family	100 to recombinase family protein (*Staphylococcus*)
J12	10716	11276	187	1E−124	None detected	100 to hypothetical protein (*Staphylococcus*)
J13	11884	12369	162	2E−112	None detected	100 to hypothetical protein (*Staphylococcus*)
J14	12632	13309	226	5E−166	NlpC/P60 family; the function of this domain is unknown; it is found in several lipoproteins	100 to hypothetical protein (*Staphylococcus*)
PSM	13578	13712	45	1E−22	*Staphylococcus* hemolytic protein	100 to beta class phenol-soluble modulin
J16	13944	14054	37	4E−17	DUF2648 superfamily; protein of unknown function	100 to multiple species; DUF2648 domain-containing protein (*Staphylococcus*)
J17	14064	15560	499	0	NADB_Rossmann superfamily; MqO malate:quinone oxidoreductase	100 to malate dehydrogenase:quinone (*S. capitis*)
J18	15780	16118	113	6E−75	DNA binding transcription regulator	100 to transcriptional regulator HXIR family (Staphylococcus caprae)
RepA	17825	18760	312	0	Replication initiator protein A (RepA) N terminus family; DNA replication initiator in plasmids	100 to replication initiator protein A (*Staphylococcus*)
J20	19190	19957	256	1E−178	Polar chromosomal segregation protein	100 to DUF536 binding domain (*Staphylococcus*)
J21	20132	20734	201	2E−140	NADB Rossmann superfamily; PRK07578 short-chain dehydrogenase	100 to short-chain dehydrogenase (bacteria)
J22	21220	21894	225	7E−165	DDE_Tnp_IS240 superfamily; Rve transposase	100 to IS*6*-like element IS*257* family transposase
*nsjF*	22148	22855	236	6.00E−119	ABC-type multidrug transport system, ATPase component (defense mechanisms)	75 to Lan protection ABC transporter ATP binding subunit in Staphylococcus succinus
*nsjE*	22857	23603	249	4E−85	Lantibiotic protection ABC transporter permease subunit, MutE/EpiE family; ABC-2 membrane superfamily	61.29 to hypothetical protein BU069_09230 in *S. succinus*
*nsjG*	23600	24337	246	1E−73	Lantibiotic protection ABC transporter permease subunit, MutG family; ABC-2 membrane superfamily	52.92 to hypothetical protein in *S. succinus*
*nsjB*	24362	27277	972	8E−90	Lantibiotic dehydratase C-terminal, thiopeptide-type bacteriocin biosynthesis domain	30.11 to lantibiotic dehydratase Lactobacillus bombicola
*nsjT*	27450	29000	517	2E−120	MdIB: ABC-type multidrug transport system, ATPase and permease component (defense mechanisms)	40.95 to ABC transporter ATP-binding protein *L. bombicola*
*nsjC*	28993	30222	410	2E−40	LanC is the cyclase enzyme of lanthionine synthetase; LanC-like superfamily	29.31 to lanthionine synthetase family protein (Bacillus nakamurai)
*nisJ*	30263	30445	61	1E−09	Structural gene; lantibiotic precursor in gallidermin/nisin family	62.5 to nisin H structural protein (Streptococcus hyointestinalis)
*nsjP*	30565	31905	447	2E−58	Peptidase S8 family domain in lantibiotic-specific proteases	32.58 to peptidase S8 (Bacillus endophyticus)
J31	31962	32357	132	7E−88	None detected	99.24 to hypothetical protein (S. epidermidis)
J32	32449	33057	203	4E−144	Serine recombinase revolvase invertase superfamily; PinE	100 to multiple species; recombinase family protein (*Staphylococcus*)
J33	33277	33477	67	1E−39	Predicted transcriptional regulator; COG3905 superfamily	100 to plasmid replication-associated protein (S. epidermidis)
ParA	33483	34277	265	0	ParA family chromosomal segregation and plasmid partition; cellulose biosynthesis protein BcsQ	99.62 to ParA family protein (S. epidermidis)
J35	34343	34882	180			No significant similarity found
RepA	35097	36089	331	0	DNA replication initiator of plasmids; HTH superfamily	99.7 to replication initiator protein A (*S. capitis*)
J37	36119	36820	234	1E−173	Putative transposase (InsQ) DNA-binding domain; OrfB_Zn_ribbon superfamily	100 to transposase (*S. capitis*)
J38	36827	37102	92	8E−60	None detected	100 to hypothetical protein EQ811_12225 (*S. capitis*)
J39	37705	37857	51	1E−27	None detected	100 to transposase (S. aureus)
RepB	38075	38932	286	0	COG5527 superfamily	99.65 to RepB family plasmid initiator protein (*Staphylococcus*)
J41	39215	39664	150	2E−99	None detected	100 to hypothetical protein (*Staphylococcus*)
J42	39867	40565	233	3E−169	None detected	98.28 to hypothetical protein (*Staphylococcus*)
J43	40667	41023	119	5E−80	None detected	100 to hypothetical protein (*Staphylococcus*)
J44	41124	41618	165	1E−107	Asp_carb_tr superfamily; pyrimidine biosynthesis	99.38 to aspartate carbamoyltransferase (S. epidermidis)
J45	41674	41847	58	2E−22	None detected	93.48 to molybdopterin biosynthesis protein MoaB
HTH	41985	42665	227	3E−161	HTH superfamily	99.12 to “winged” HTH transcription regulator (S. epidermidis)
J47	42777	44150	458	0	Multidrug resistance MFS family permease; transport and metabolism	99.78 to MFS transporter (S. epidermidis)
J48	44846	45340	165	3E−109	None detected	98.78 to hypothetical protein (S. epidermidis)
J49	45337	46101	255	6E−176	None detected	100 to hypothetical protein (S. epidermidis)
HTH	46186	46455	90	2E−58	HTH XRE superfamily	100 to HTH transcription regulator (Auricoccus indicus)
J51	47042	47155	38	1E−15	None detected	100 to hypothetical protein UF66_0802 (Staphylococcus cohnii subsp. *cohnii*)
J52	48210	48476	89	8E−57	None detected	98.86 to multispecies hypothetical protein (*Staphylococcus*)
J53	48623	49843	407	0	None detected	100 to hypothetical protein (*S. capitis*)

a aa, amino acid. HTH, helix-turn-helix; XRE, xenobiotic response element; MFS, major facilitator superfamily.

### Other genes contained in the *S. capitis* APC 2923 draft genome.

In addition to the nisin J cluster, BAGEL4 and antiSMASH3.0 also highlighted a small gene cluster containing the *lanB* and *lanC* genes and a gene encoding a peptide with 93% identity to the gallidermin family in *S. capitis* APC 2923. These were located on a different contig from that of the nisin J gene cluster, and this mass was not detected from either the colony or purified cell free supernatants.

### Purification and predicted structure of nisin J.

**FIG 3:**
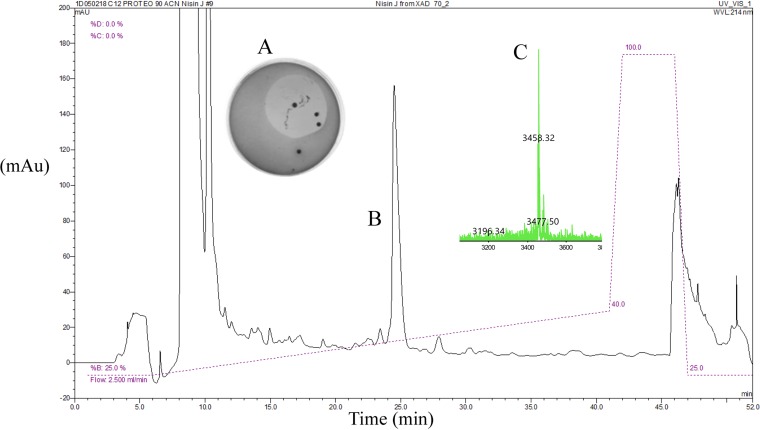
Purification of nisin J from *S. capitis* APC 2923 grown in XAD-BHI. (A) Original overlay plate where antimicrobial activity of the *S. capitis* APC 2923 strain was detected. (B) The RP-HPLC profile shows a peak at HPLC-active fraction of 24.5 minutes, which correlates with where pure nisin J elutes. (C) MALDI-TOF MS of active fraction. mAu, milli-arbitrary units.

**FIG 4:**
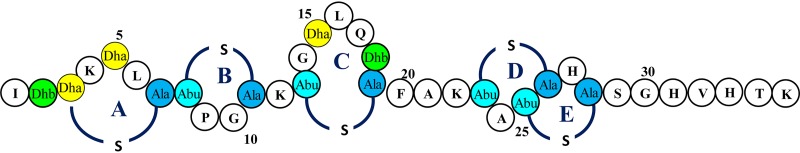
Proposed structure of the novel nisin variant nisin J. Residues are represented by the single-letter code. Posttranslational modifications are indicated as follows: Dha, dehydroalanine; Dhb, dehydrobutyrine; Abu, 2-aminobutyric acid; Abu-S-Ala, 3-methyllanthionine.

Nisin J was purified in four steps using Amberlite XAD-16N solid-phase extraction (SPE), SP Sepharose cation exchange, C_18_ SPE, and reversed-phase high-performance liquid chromatography (HPLC). Antimicrobial activity correlated with the most dominant peak eluting at 24.5 min in the HPLC chromatogram, and matrix-assisted laser desorption ionization–time of flight mass spectrometry (MALDI-TOF MS) revealed that the corresponding fractions had a mass of 3,458 Da (Fig. 3). This correlates with the predicted mass of the putative nisin J bacteriocin (following subsequent dehydration and ring formation reactions) as calculated from the draft genome sequence. Fractions deemed pure by MALDI-TOF MS were combined and lyophilized to give a yield of 3.00 mg/liter. Given that nisin J is a natural nisin variant with demonstrable conservation between key structural amino acids common to all natural nisin variants, it is predicted that the structure will be in line with those of other lactococcal nisins, as shown in Fig. 4.

### Comparing the activities of purified nisins A, Z, and J.

The spectrum of activity of pure nisin A, nisin Z, and nisin J, by means of a well diffusion assay (WDA), was performed on several target indicator strains. Nisin J was more active than nisin A against 12 of the 13 strains tested, while nisin J was more active than nisin Z for 7 of the target strains tested, including Corynebacterium xerosis, MRSA, Streptococcus uberis, and S. aureus (Table 2). However, in an MIC assay using L. lactis HP as the indicator, no difference was observed between nisins A, Z, and J, with all exhibiting MICs of 32 nM.

**TABLE 2:** Inhibition spectra of purified peptides of nisins A, Z, and J against indicator strains using well diffusion assays and expressed as the area of the zone of inhibition

Target microorganism	Strain	Area of zone of inhibition^*a*^ (mm^2^) for nisin:		
		A	Z	J
*Corynebacterium xerosis*	DPC 5629	51.5	66.2	133.8
*Cutibacterium acnes*	LMG 16711	537	587.5	469
*Lactobacillus delbrueckii* subsp. *bulgaricus*	LMG 6901	555.7	672.7	651.44
*Lactococcus lactis* subsp. *cremoris*	HP	241.9	325.8	363
*Listeria monocytogenes*	WSLC 112	60	73	37
*Enterococcus faecium*	APC 852	93.2	120.6	170.9
*Enterococcus faecalis*	ATCC 19433	101.2	120.8	102.97
Methicillin-resistant *Staphylococcus aureus*	DPC 5645	77	115.9	135.8
*Staphylococcus aureus*	DPC 7016	109.4	143.4	153.1
*Staphylococcus epidermidis*	DPC 5990	136.8	180.3	159.5
*Staphylococcus simulans*	APC 3482	148.7	197.1	395.5
*Streptococcus agalactiae*	ATCC 13813	174.4	221.7	136.8
*Streptococcus uberis*	DPC 5344	98.5	153.9	248.8

a Calculated as the area of zone of inhibition (πr^2^) − area of well (πr^2^) in millimeters. Assays were carried out in duplicate; mean zone areas shown.

### The nisin J-producing strain is cross-immune to nisin A and H but not to nisin U producers.

Cross-immunity assays were performed to investigate whether the nisin J-, A-, H-, and U-producing strains were cross-immune to one another (Table 3). No zones were observed between nisins A, H, and J, indicating that these producing strains are all cross-immune. However, a zone was observed from the nisin J-producing strain against the nisin U producer (*S. uberis* strain 42), demonstrating that the strain is sensitive to nisin J.

**TABLE 3:** Cross-immunity of nisin A-, U-, H-, and J-producing strains using well diffusion assays and expressed as the area of the zone of inhibition

Target organism	Strain	Nisin produced	Area of zone of inhibition^*a*^ (mm^2^) against nisin:			
			A	U	H	J
Lactococcus lactis	NZ9700	A	0	0	0	0
Streptococcus uberis	42	U	0	0	0	85
Streptococcus hyointestinalis	DPC 6484	H	0	0	0	0
Staphylococcus capitis	APC 2923	J	0	0	0	0

a Calculated as the area of zone of inhibition (πr^2^) − area of well (πr^2^) in millimeters. Values are the means from triplicate assays. 0, no zone observed.

### Not all *S. capitis* strains contain a nisin-like gene cluster.

The *nisJ* structural gene was amplified from nine antimicrobial-producing *S. capitis* strains isolated from human skin in a previous study by our group (5). Two of the nine *S. capitis* strains (APC 2918 and APC 2934) did not contain the *nisJ* structural gene. The other seven *S. capitis* strains tested positive for the *nisJ* structural gene, correlating with findings from our earlier study which found these strains to be cross-immune and to possess the same pulsotype, indicating that they were the same strain or very closely related strains and were therefore most likely producing the same bacteriocin (5). These 7 strains were isolated from 4 different subjects, indicating that the same pulsotype is shared across a number of individuals, implying that the ability to produce nisin J may be a dominant feature and thus an ecological advantage for this *S. capitis* strain.

### The nisin J gene cluster resides on a plasmid.

**FIG 5:**
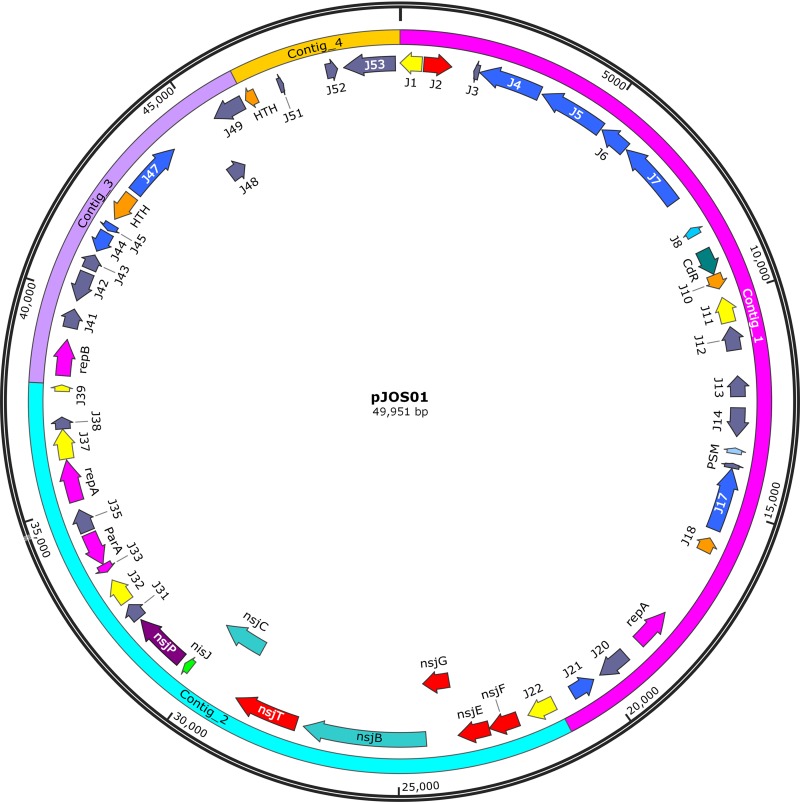
Plasmid map of pJOS01 draft sequence created on SnapGene version 2.3.2 (GenBank accession number MN602039).

Analysis of the *S. capitis* APC 2923 contig harboring the nisin J gene cluster identified the presence of a plasmid replication protein A (RepA) and other plasmid replication-associated proteins, suggesting that it was of plasmid origin. Plasmid DNA was readily obtained from *S. capitis* APC 2923 using a commercially available plasmid maxi kit (data not shown). Short-read sequencing was performed on the plasmid DNA using the Illumina MiSeq platform to approximately 200-fold coverage. *De novo* assembly resulted in four contigs (Fig. 5), with a combined size of 49,951 bp. A plasmid map of pJOS01 (GenBank accession number MN602039) shows all of the genes encoding immunity and the biosynthetic machinery for nisin J (*nsjFEG*, *nsjB*, *nsjT*, *nsjC*, *nisJ*, and *nsjP*) reside on one of the contigs, supporting the plasmid association of the nisin J gene cluster (Fig. 5). Furthermore, three genes encoding plasmid replication functions (RepA and RepB) as well as genes encoding other nonessential plasmid-associated roles were present on the other contigs (Fig. 5 and Table 1). Restriction digestion with EcoRI yielded a profile comparable to the virtual digestion of the generated plasmid sequence, supporting the predicted size of ∼50 kb (data not shown). Subsequent analysis revealed a GC content of ∼28%, which is considerably lower than that of *S. capitis* chromosomal DNA (32 to 33%), a characteristic that has been observed for plasmids of many Gram-positive species (28).

### Nisin J exhibits resistance to NSR.

**FIG 6:**
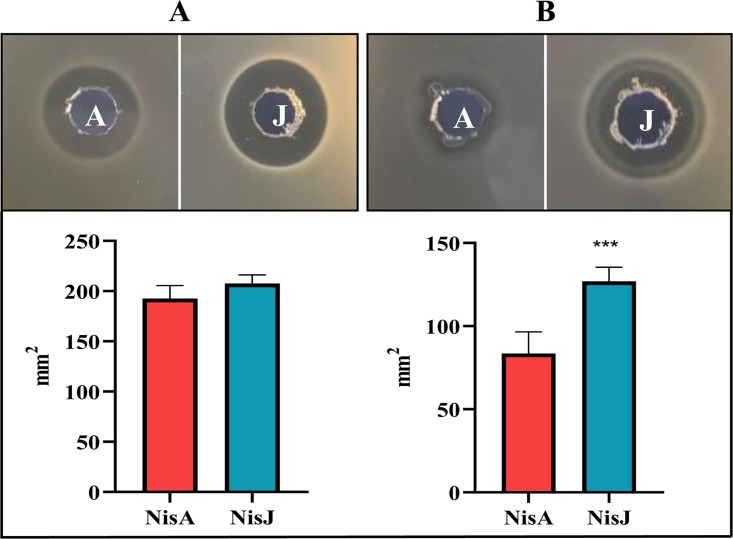
Activity of cell-free supernatant of nisin J- and A-producing strains as observed in WDA against MG1614 (NSR^−^) (A) and MG1614/pNP40 (NSR^+^) (B). The assay results are representative of triplicate experiments. The results reveal no significant differences in zones of inhibition against NSR^−^ (*P* value of 0.1701) (bar graph in panel A) but show a significant difference (***) against NSR^+^ (*P* value of 0.0001) (bar graph in panel B).

Deferred antagonism assays using L. lactis subsp. diacetylactis DRC3 (nisin resistance protein positive [NSR^+^]) as a target indicator strain revealed that nisin J is partially resistant to NSR (result not shown). To establish if nisin J had increased inhibitory activity against NSR compared to that of nisin A, further WDAs were conducted using the NSR^+^ and NSR^−^ strains L. lactis MG1614/pNP40 and L. lactis MG1614, respectively. While the inhibition zone of the nisin J producer is slightly decreased against the NSR-positive strain compared to the NSR-negative strain, it appears that nisin J is more active than nisin A and may be less susceptible to the proteolytic effects of NSR (Fig. 6A), which was also demonstrated in agarose assays (Fig. 6B). The analysis revealed a significant difference in the zones of inhibition between nisin A and nisin J against an NSR^+^ strain (MG1614/pNP40), with a *P* value of 0.0001 compared to zone sizes against an NSR^−^ strain (MG1614), where no statistical difference (*P* = 0.1701) was observed (these data support Fig. 6).

## DISCUSSION

As the burden of antibiotic resistance increases globally, there is an urgent need for novel therapeutic options. In addition to the well-established use of nisin as a food preservative, many studies have focused on using nisin against drug‐resistant pathogens in clinical or veterinary settings due to its high potency and multiple mechanisms of action (10–12). Nisin J is a novel nisin variant and the first such variant reported from a *Staphylococcus* species. A combination of whole-genome sequencing of *S. capitis* APC 2923 and peptide purification resulted in the identification of this broad-spectrum lantibiotic. The nisin J-producing *S. capitis* strain was isolated from the toe web space, an area associated with high microbial load. This suggests that the production of a broad-spectrum bacteriocin confers an advantage on this strain over competing commensal skin flora, as was also observed by O’Sullivan and colleagues (5) when four of the twenty subjects screened in the study exhibit the same pulsotype. The residence of the nisin J gene cluster on a plasmid is significant in that it may facilitate its dissemination to other skin microbes.

As mentioned previously, nisin J has eight amino acid changes and one extra amino acid near the C-terminal end compared to nisin A. Interestingly, six of the eight changes are unique compared to natural nisin variants. Natural nisin variants are tolerant to some amino acid changes at the N terminus, with Ile4 being the most commonly substituted amino acid. Nisin J contains an Ile4Lys substitution which is also seen in nisins P, U, U_2_, and O_1–3_ but remains unchanged in lactococcal nisins (A, Z, F, and Q) and nisin H. Nisin J differs most from other natural nisin variants in the center and at the C terminus of the peptide, which could be key to nisin J’s enhanced activity. At the center of nisin J, amino acid positions 17 to 21, there are 4 amino acids that differ compared to nisin A. It contains a Met17Gln substitution which is unique, as all other natural nisin variants that demonstrate antimicrobial activity have Met at this position. The Gly18Thr change is also interesting, as it is observed in nisins H, U, U_2_, P, and O_1–3_ and is proposed to be modified to dehydrobutyrine (Dhb), in light of the dehydration observed in a M17Q-G18T derivative of nisin Z (29). At position 20, nisin J has a highly hydrophobic residue, phenylalanine, compared to the polar asparagine in nisin A. Li et al. (30) found that extending the C terminus of nisin improves both its ability to permeate membranes and its inhibitory potential against Gram-negative bacteria. Therefore, nisin J’s longer C terminus (compared to other nisin variants) could be more attracted to negatively charged cell membranes resulting in enhanced membrane insertion, which may be responsible for its broader host range. The skin origin of this nisin J producer suggests that its exposure to many competitors from the external environment may be responsible for the greater variation in the structure of nisin J.

Analysis of the nisin J gene cluster identified several key features associated with bacteriocin operons. These include a structural gene (*nisJ*), 2 genes associated with enzymatic modification (*nsjB* and *nsjC*), a gene involved in transport (*nsjT*), and immunity genes (*nsjFEG*) (Table 1 lists the identity and functions of features of the nisin J gene cluster). The arrangement of genes in the nisin J gene cluster differs from that of other nisin operons. Interestingly, the only conservation of gene order throughout all operons of natural nisin variants is *lanBTC*. Similarities in the structural peptides of different nisin variants from different origins indicate the possibility that an evolutionary link exists between lactococcal, streptococcal, *Blautia*, and now, staphylococcal species, a link previously mentioned by O’Connor et al. (27) with reference to streptococcal and lactococcal species. A dendrogram based on the primary structures of all known natural variants highlights the genetic relatedness between the nisin-producing species and further suggests the likelihood of this evolutionary link. The FEG locus is present in lantibiotic systems other than nisin, including subtilin (31) and epidermin (32), and has been linked to transport, immunity, and defense (33). Inactivation of these genes in the nisin A gene cluster decreased nisin production and immunity, confirming their role in immunity (34). Although the *nsjFEG* genes are present in the nisin J gene cluster, the absence of a specific immunity gene, *nsjI*, as well as the absence of an expression regulatory system, *nsjRK*, could explain why nisin J immunity mechanisms appear to be less able to protect the cell. It also further supports the finding that the producing strain was more sensitive to its own purified nisin J peptide than was a nisin A producer with a specific nisin immunity determinant.

The production of lantibiotics such as gallidermin and epidermin is associated with increased release of lipids and ATP and protein excretion, which are indicators of cell membrane damage (35). Thus, the production of these lantibiotics has been deemed a “burden” to staphylococci that produce them; therefore, the incomplete lantibiotic gene cluster, having only the *lanB* and *lanC* genes present, may be either an evolutionary feature of *S. capitis* genomes or may be an incomplete cluster of lantibiotic biosynthetic genes previously shown to occur in many microbes (35).

As previously discussed, the nisin J gene cluster resides on a plasmid, inviting the speculation that *S. capitis* acquired its antimicrobial ability through horizontal gene transfer. Indeed, residence on mobile genetic elements is a feature of natural nisin variants, as observed with nisins A and H, and may explain their presence in many different species.

Purification of nisin J resulted in a peptide with a mass of 3,458 Da. The mass of nisin J was predicted to be 3,622 Da, where the difference between predicted and observed masses can be accounted for by 9 dehydration reactions (−18 Da per loss of water residue) involved in the formation of lanthionine and β-methyllanthionine bridges (36). The predicted peptide structure was based on the nisin A template, with a lanthionine bridge likely to occur between Ser3 and Cys7 and four β-methyllanthionine bridges between Thr8 and Cys11, Thr13 and Cys19, Thr23 and Cys26, and Thr25 and Cys28.

True to all nisin variants, nisin J is a broad-spectrum lantibiotic with inhibitory activity similar to that of nisins A and Z, as can be seen in Table 2, inhibiting a wide range of bacterial genera with greater inhibition of staphylococcal targets than with nisins A and Z. This suggests that the nisin J-producing *S. capitis* strain may have naturally evolved to produce a nisin peptide with enhanced activity against other staphylococci in the skin microbiota (Table 2). Nisin J-, A-, and H-producing strains are immune to nisin peptides J, A, H, and U; however, the nisin U-producing strain is not immune to nisin J (Table 3). This may be due to the lack of the *nsjI* immunity gene in the nisin J cluster.

The nisin resistance protein (NSR) is a protease which cleaves nisin A at Ser29, significantly reducing the activity of the peptide. Employing a bioengineering strategy, Field et al. (37) demonstrated that the substitution of residues 29 and 30 with proline and valine, respectively (derivative designated S29PV), rendered the peptide resistant to proteolytic digestion by NSR. In this study, we found that the nisin J producer displays a higher resistance to NSR proteolytic enzymes than does nisin A, which is possibly due to a glycine residue at position 30 instead of the isoleucine as found in nisin A. Interestingly, a study carried out by Simões et al. (38) involving a multidrug-resistant *S. capitis* clone, NRCS-A, a major pathogen involved in sepsis in preterm neonates, demonstrated the presence of an NSR-encoding gene. PCR analysis failed to detect the presence of any *nsr* gene in any nisin J-producing *S. capitis* strain from our previous study (5).

Nisin J may have evolved to be more potent against specific competing organisms in a particular niche environment such as the skin. Employing a bioengineering strategy, Rink et al. (39) demonstrated that the replacement of residues I, S, and L at positions 4, 5, and 6 in nisin A with the residues K, S, and I, respectively, resulted in enhanced bioactivity. Notably, the residues K-S-L are naturally present in nisin J at the same positions. In a separate bioengineering study, Kuipers et al. (29) generated a novel nisin variant (M17Q/G18T) exhibiting enhanced bioactivity. It is interesting that both of these mutations are naturally present in nisin J. Furthermore, Field et al. (40) reported that a nisin A derivative, M21A, demonstrated enhanced bioactivity. Remarkably, alanine is naturally present at position 21 in nisin J.

In conclusion, we have identified a new natural nisin variant, nisin J, produced by *S. capitis* APC 2923, which was isolated from the human skin microbiota. Nisin J represents the first nisin variant isolated from *Staphylococcus* species and the first to demonstrate partial recalcitrance to NSR. Indeed, the enhanced activity of nisin J compared to that of nisin A and Z as observed against all staphylococcal strains utilized in this study is notable. The production of bacteriocins such as nisin J from skin bacteria highlights the potential of bacterial strains of skin origin to be used as live biotherapeutics.

## MATERIALS AND METHODS

The antimicrobial-producing strain *S. capitis* APC 2923 was isolated in a previous screening study of the human skin microbiota by our group (5).

### Bacterial strains and culture conditions.

The growth conditions of the bacterial strains used in this study are listed in Table 4. Anaerobic conditions, where appropriate, were attained using anaerobic jars and Anaerocult A gas packs (Merck, Darmstadt, Germany).

**TABLE 4:** Growth conditions of the bacterial strains used in this study

Species	Strain^*a*^	Growth conditions		
		Temp (°C)	Atmosphere^*b*^	Growth medium(a)^*c*^
Corynebacterium xerosis	DPC 5629	37	Aerobic	BHI
Cutibacterium acnes	LMG 16711	37	Anaerobic	mRCM and RCA
Enterococcus faecalis	ATCC 19433	37	Anaerobic	MRS
Enterococcus faecium	APC 852	37	Anaerobic	MRS
Lactobacillus delbrueckii subsp. *bulgaricus*	LMG 6901	37	Anaerobic	MRS
Lactococcus lactis	NZ9700	30	Aerobic	GM17
Lactococcus lactis subsp. *cremoris*	HP	30	Aerobic	GM17
Lactococcus lactis	MG1614	30	Aerobic	GM17
Lactococcus lactis	MG1614/pNP40	30	Aerobic	GM17
Lactococcus lactis subsp. *diacetylactis*	DRC3	30	Aerobic	GM17
Listeria monocytogenes	WSLC 1211	37	Aerobic	BHI
MRSA^*d*^	DPC 5645	37	Aerobic	BHI
Staphylococcus aureus	DPC 7016	37	Aerobic	BHI
Staphylococcus capitis	APC 2923	37	Aerobic	BHI
Staphylococcus epidermidis	DPC 5990	37	Aerobic	BHI
Staphylococcus simulans	APC 3482	37	Aerobic	BHI
Streptococcus agalactiae	ATCC 13813	37	Aerobic	BHI
Streptococcus hyointestinalis	DPC 6484	37	Anaerobic	GM17
Streptococcus uberis	DPC 5344	37	Aerobic	BHI
Streptococcus uberis	Strain 42	37	Anaerobic	GM17

a ATCC, American Type Culture Collection; APC, APC Microbiome Ireland Culture Collection; DPC, Teagasc Culture Collection; WSLC, Weihenstephan *Listeria* Collection; LMG, Laboratorium voor Microbiologie.

b Anaerobic conditions, where appropriate, were achieved through the use of anaerobic jars and Anaerocult A gas packs (Merck, Darmstadt, Germany).

c MRS, de Man-Rogosa-Sharpe; mRCM, modified reinforced *Clostridium* medium (made following the ATCC medium: 2107 modified reinforced clostridial agar/broth [prereduced] protocol); RCA, reinforced *Clostridium* agar; BHI, brain heart infusion; GM17, 0.5% glucose added to M17 agar.

d MRSA, methicillin-resistant S. aureus.

### Draft genome sequence of *S. capitis* APC 2923 and *in silico* analysis of the nisin J gene cluster.

Bacterial DNA was extracted using the GenElute kit, as described by the manufacturer (Sigma-Aldrich Ireland Limited, Arklow, County Wicklow, Ireland), and was prepared for sequencing following the Nextera XT DNA library prep reference guide (Illumina, Inc.). A Qubit 3.0 fluorometer (Thermo Fisher Scientific, MA) was used for DNA quantification. Sequencing was performed at the Teagasc/APC Microbiome Ireland Sequencing facility, Teagasc Food Research Centre, Moorepark, Fermoy, County Cork, Ireland. In total, 94 contigs, including 16 large contigs, were revealed by *de novo* assembly using SPAdes (version 3.10.0). A total of 2,453 open reading frames (ORFs) and 60 tRNAs were detected and subsequently annotated using Prokka (version 1.11). The online tools Bacteriocin GEnome mining tooL (BAGEL4) and antiSMASH 3.0 were employed to identify bacteriocin operons/gene clusters in the genomes of interest, and by combining these software programs with the ARTEMIS genome viewer, the presence of the nisin J gene cluster was confirmed.

### Evolutionary links between natural nisin variants.

The European Bioinformatics Institute toolkit (https://www.ebi.ac.uk/services) was used to investigate the evolutionary relationships between the nisin structural variants. A multiple-sequence alignment was generated using MUSCLE (version 3.8) and visualized on a neighbor-joining tree without distance corrections. This tree was visualized using the ggtree package (version 1.10.5) in R (version 3.4.4).

### Purification of the antimicrobial produced by *S. capitis* APC 2923.

To purify the antimicrobial produced by *S. capitis* APC 2923, the culture was grown in a shaking 37°C incubator overnight in 1,800 ml of brain heart infusion (BHI) which had been passed through an XAD column to remove hydrophobic peptides before autoclaving (XAD-BHI). The culture supernatant was applied to an Econo-Column containing 60 g Amberlite XAD-16N beads (Sigma Aldrich, Arklow, Co. Wicklow, Ireland). The column was then washed with 350 ml of 30% ethanol, and the antimicrobial activity was eluted with 70% propan-2-ol (IPA) containing 0.1% trifluoroacetic acid (TFA) (Sigma Aldrich). The IPA was removed from the active column eluent and the pH adjusted to 4.4 with 7.5 N NaOH. The sample was then applied to an Econo-Column containing 90 ml SP Sepharose beads preequilibrated with 20 mM sodium acetate buffer (pH 4.4) (buffer A). The column was washed with 50 ml of buffer A and the antimicrobial activity eluted in 250 ml buffer A containing 1 M NaCl. The salt-containing eluent was applied to 60 ml of a 10-g C_18_ solid-phase extraction (SPE) column (Phenomenex, Cheshire, United Kingdom) preequilibrated with methanol and water. The column was washed with 60 ml of 25% ethanol, and nisin was eluted in 60 ml IPA (0.1% TFA), which was subjected to reversed-phase high-performance liquid chromatography (RP-HPLC). The sample was applied to a semipreparative Proteo Jupiter (250 mm [length] by 10 mm [inside diameter], 90 Å [pore size], 4 μm [particle size]) RP-HPLC column (Phenomenex) running a gradient of 25 to 40% acetonitrile and 0.1% TFA, where buffer B was 90% acetonitrile and 0.1% TFA. The resulting eluent was monitored at 214 nm, and fractions were collected at 1-min intervals. Column eluents and HPLC fractions were assayed for antimicrobial activity by well diffusion assays (WDAs), according to the method of Parente and Hill (41), using L. delbrueckii subsp. bulgaricus LMG 6901 as the target organism. Column eluents and HPLC fractions displaying antimicrobial activity were assayed for the nisin J molecular mass by matrix-assisted laser desorption ionization–time of flight mass spectrometry (MALDI-TOF MS) on an Axima TOF^2^ MALDI-TOF MS in positive-ion reflectron mode (Shimadzu Biotech, Manchester, United Kingdom). Fractions containing pure nisin J were pooled and lyophilized in a Genevac lyophilizer (Suffolk, United Kingdom). Pure nisin A peptide was prepared from L. lactis NZ9700 as described for nisin J but excluding the SP Sepharose step. Nisin Z pure peptide was sourced from Handary (Fleurus, Belgium).

### Comparison of the inhibitory spectra of nisins A, Z, and J.

Pure nisins A, Z, and J were resuspended in RNase-free water to a final concentration of 1 mg/ml and subsequently assayed by WDA against a range of target indicator strains (Table 2). Zone diameters were measured in millimeters using Vernier calipers (DML-Digital Micrometers Ltd., Sheffield, United Kingdom) and recorded in Table 2 as area of the zone (π*r*^2^) minus the area of the well (π*r*^2^) in millimeters.

### MIC determinations.

MICs were determined in triplicate from pure nisins A, Z, and J against approximately 1 × 10^5^ CFU/ml of the target indicator strain Lactococcus lactis subsp. cremoris HP using 96-well microtiter plates (Sarstedt, Co. Wexford, Ireland) and using a Libra S2 colorimeter (Biochrom Ltd., Cambridge, United Kingdom) to measure the optical density at 600 nm (OD_600_) of the indicator strains. Peptide concentrations of 4× the test concentration (2,048 nM) were prepared in 400 μl RNase-free and DNase-free water. One hundred microliters of growth medium was added to all wells of the 96-well plate. One hundred microliters of 4× concentration was added to the first well, and subsequently, 2-fold serial dilutions were carried out. MIC readings were taken after 16 h at 30°C. The MIC was recorded as the lowest concentration of lantipeptide where no growth of the indicator was observed (42).

### Cross-immunity of nisin J-producing *S. capitis* APC 2923 to other nisin-producing strains.

To investigate if the nisin J-producing *S. capitis* APC 2923 strain was immune to other nisin-producing cultures (L. lactis NZ9700 producing nisin A, Streptococcus hyointestinalis DPC 6484 producing nisin H, and *S. uberis* strain 42 producing nisin U), cross-immunity assays were performed based on the WDA method, whereby each strain was tested as an indicator and a producer (43).

### Determining if the nisin J structural gene is unique to *S. capitis* APC 2923.

To determine if the nisin J structural gene was present in other *S. capitis* strains isolated from the study by O’Sullivan et al. (5), oligonucleotide primers designed to specifically amplify the nisin J structural gene (*nisJ* F, 5′-ACTTTATAACTAAGATTAGC-3′, and *nisJ* R, 5′-TCGCTTTATTATTTAGTATGCACG-3′) were used in a PCR under the following conditions: initial denaturation, 94°C for 5 min; 35 cycles of 94°C for 40 s, 52°C for 30 s, and 72°C for 1 min; and a final extension 72°C for 10 min. Sequencing was conducted by Genewiz (Essex, United Kingdom). Sequencing data were analyzed employing the Lasergene 8 software (DNAStar, Inc., Madison, WI) and subsequently input into the ExPASy online translate tool (https://web.expasy.org/translate/) to translate the nucleotides into amino acid sequences.

### Sequence analysis of the nisin J plasmid pJOS01.

To confirm that the nisin J gene cluster was plasmid associated, the plasmid DNA of *S. capitis* APC 2923 was extracted using the Plasmid maxi kit (Qiagen, Hilden, Germany), according to the manufacturer’s instructions following an adapted user-developed protocol specific to staphylococcal species (https://www.qiagen.com/ie/resources/resourcedetail?id=82ddd661-fbab-4d35-819c-defd6269fc64&lang=en), using lysostaphin (Sigma-Aldrich Ireland Limited, Arklow, County Wicklow, Ireland). The resulting DNA extract was sequenced by Illumina MiSeq technology (2 × 250-bp paired-end reads; GenProbio, Parma, Italy). *De novo* sequence assemblies and automated gene calling were performed using the MEGAnnotator pipeline (44) and assessed for predicted tRNA genes via transcend-SE version 1.2.1 (45). Predicted open reading frames (ORFs) were determined via Prodigal version 2.6 and Genemark.hmm (46). A BLASTP (47) analysis was performed to assign functional annotations to the predicted ORFs (https://blast.ncbi.nlm.nih.gov/Blast.cgi) (Table 1). PlasmidFinder (version 2.0) was employed to confirm that the generated assembled contigs were plasmid sequences based on the identification of Rep proteins. SnapGene version 2.3.2 was employed to generate a map of the plasmid harboring the *nisJ* gene cluster (designated pJOS01 here). In addition to the sequence data analysis to confirm the plasmid association of the nisin J cluster, PCR-based analysis was undertaken using the plasmid DNA extract as the template. Oligonucleotide primers designed to specifically amplify the nisin J structural gene (*nisJ* F, 5′-ACTTTATAACTAAGATTAGC-3′, and *nisJ* R, 5′-TCGCTTTATTATTTAGTATGCACG-3′) were used in a PCR using Phusion Green Hot Start II high-fidelity PCR master mix with the following conditions: initial denaturation, 98°C for 5 min; 30 cycles of 98°C for 10 s, 52°C for 30 s, and 72°C for 15 s; and a final extension of 72°C for 10 min. Validation of the amplicon was performed by Sanger sequencing of the generated product (Source BioScience, Waterford, Ireland). Furthermore, restriction digestion of the plasmid DNA was carried out using EcoRI in 10× CutSmart buffer (New England BioLabs, Herts, United Kingdom).

### Investigation for the presence of nisin-resistant determinants in *S. capitis* APC 2923.

To determine if the gene encoding the nisin resistance protein (NSR) was present in *S. capitis* APC 2923 and the 7 other *S. capitis* isolates previously identified from the O’Sullivan et al. study (5), PCR was employed using the primers and reaction conditions described by Simões et al. (38). To determine if the nisin J-producing *S. capitis* strain APC 2923 was cross-immune or sensitive to NSR-producing strains, bioassays were carried out by spotting 10 μl of the nisin J overnight culture onto 1.5% BHI agar (Merck, Darmstadt, Germany). Following overnight incubation at 37°C, the plates were then overlaid with soft (0.75%) GM17 agar (BD Difco Trafalgar Scientific Ltd., Leicester, United Kingdom) seeded with 0.25% of an overnight culture of the NSR-positive strain L. lactis subsp. diacetylactis DRC3. To directly compare the resistance levels of nisin A and nisin J to NSR, WDAs were carried out as previously described (43), employing L. lactis MG1614/pNP40 (NSR-positive strain) and L. lactis MG1614 (NSR-negative strain) as target indicators. All lactococcal NSR indicator strains were grown aerobically overnight at 30°C. Agarose assays were subsequently performed as outlined in reference 42. Data obtained from the agarose assays were subjected to normality tests prior to statistical analysis using the GraphPad Prism software (version 8.2.1). *P* values were calculated using an unpaired *t* test.

### Data availability.

The plasmid map of pJOS01 has been deposited in GenBank under accession number MN602039. This whole-genome shotgun project has been deposited at DDBJ/ENA/GenBank under the accession number WHVU00000000. The version described in this paper is version WHVU01000000.1.

## References

[1] Sender R, Fuchs S, Milo R 2016 Revised estimates for the number of human and bacteria cells in the body. PLoS Biol 14:e1002533. doi:10.1371/journal.pbio.1002533. 27541692 PMC4991899

[2] Cameron DR, Jiang JH, Hassan KA, Elbourne LDH, Tuck KL, Paulsen IT, Peleg AY 2015 Insights on virulence from the complete genome of staphylococcus capitis. Front Microbiol 6:980. doi:10.3389/fmicb.2015.00980. 26441910 PMC4585213

[3] Sugai M, Fujiwara T, Akiyama T, Ohara M, Komatsuzawa H, Inoue S, Suginaka H 1997 Purification and molecular characterization of glycylglycine endopeptidase produced by Staphylococcus capitis EPK1. J Bacteriol 179:1193–1202. doi:10.1128/jb.179.4.1193-1202.1997. 9023202 PMC178816

[4] Kumar R, Jangir PK, Das J, Taneja B, Sharma R 2017 Genome analysis of Staphylococcus capitis TE8 reveals repertoire of antimicrobial peptides and adaptation strategies for growth on human skin. Sci Rep 7:10447. doi:10.1038/s41598-017-11020-7. 28874737 PMC5585272

[5] O’Sullivan JN, Rea MC, O'Connor PM, Hill C, Ross RP 2019 Human skin microbiota is a rich source of bacteriocin-producing staphylococci that kill human pathogens. FEMS Microbiol Ecol 95:fiy241. doi:10.1093/femsec/fiy241. PMC6340406 30590567

[6] Dobson A, Cotter PD, Paul Ross R, Hill C 2012 Bacteriocin production: a probiotic trait? Appl Environ Microbiol 78:1–6. doi:10.1128/AEM.05576-11. 22038602 PMC3255625

[7] Corr SC, Li Y, Riedel CU, O'Toole PW, Hill C, Gahan CGM 2007 Bacteriocin production as a mechanism for the antiinfective activity of Lactobacillus salivarius UCC118. Proc Natl Acad Sci U S A 104:7617–7621. doi:10.1073/pnas.0700440104. 17456596 PMC1863472

[8] Cotter PD, Hill C, Ross PR 2005 Bacteriocins: developing innate immunity for food. Nat Rev Microbiol 3:777–788. doi:10.1038/nrmicro1273. 16205711

[9] de Pablo MA, Gaforio JJ, Gallego AM, Ortega E, Gálvez AM, Alvarez de Cienfuegos López G 1999 Evaluation of immunomodulatory effects of nisin-containing diets on mice. FEMS Immunol Med Microbiol 24:35–42. doi:10.1111/j.1574-695X.1999.tb01262.x. 10340710

[10] Brand AM, Smith C, Dicks L 2013 The effects of continuous in vivo administration of nisin on Staphylococcus aureus infection and immune response in mice. Probiotics Antimicrob Proteins 5:279–286. doi:10.1007/s12602-013-9141-3. 26783073

[11] Małaczewska J, Kaczorek-Łukowska E, Wójcik R, Rękawek W, Siwicki AK 2019 In vitro immunomodulatory effect of nisin on porcine leucocytes. J Anim Physiol Anim Nutr (Berl) 103:882–893. doi:10.1111/jpn.13085. 30916834

[12] Kindrachuk J, Jenssen H, Elliott M, Nijnik A, Magrangeas-Janot L, Pasupuleti M, Thorson L, Ma S, Easton DM, Bains M, Finlay B, Breukink EJ, Georg-Sahl H, Hancock R 2013 Manipulation of innate immunity by a bacterial secreted peptide: lantibiotic nisin Z is selectively immunomodulatory. Innate Immun 19:315–327. doi:10.1177/1753425912461456. 23109507

[13] Rogers LA, Whittier EO 1928 Limiting factors in the lactic fermentation. J Bacteriol 16:211–229. 16559334 10.1128/jb.16.4.211-229.1928 PMC375023

[14] Delves-Broughton J 2005 Nisin as a food preservative. Food Aust 57:525–527. 10.4315/0362-028X-57.10.874 31121693

[15] Cheigh CI, Pyun YR 2005 Nisin biosynthesis and its properties. Biotechnol Lett 27:1641–1648. doi:10.1007/s10529-005-2721-x. 16247668

[16] Cotter PD, Ross RP, Hill C 2013 Bacteriocins—a viable alternative to antibiotics? Nat Rev Microbiol 11:95–105. doi:10.1038/nrmicro2937. 23268227

[17] McAuliffe O, Ross RP, Hill C 2001 Lantibiotics: structure, biosynthesis and mode of action. FEMS Microbiol Rev 25:285–308. doi:10.1111/j.1574-6976.2001.tb00579.x. 11348686

[18] Kellner R, Jung G, Horner T, Zahner H, Schnell N, Entian K‐D, Gotz F 1988 Gallidermin: a new lanthionine‐containing polypeptide antibiotic. Eur J Biochem 177:53–59. doi:10.1111/j.1432-1033.1988.tb14344.x. 3181159

[19] Schnell N, Entian K-D, Schneider U, Götz F, Zähner H, Kellner R, Jung G 1988 Prepeptide sequence of epidermin, a ribosomally synthesized antibiotic with four sulphide-rings. Nature 333:276–278. doi:10.1038/333276a0. 2835685

[20] Kim PI, Sohng JK, Sung C, Joo H-S, Kim E-M, Yamaguchi T, Park D, Kim B-G 2010 Characterization and structure identification of an antimicrobial peptide, hominicin, produced by Staphylococcus hominis MBBL 2-9. Biochem Biophys Res Commun 399:133–138. doi:10.1016/j.bbrc.2010.07.024. 20654578

[21] Alvarez-Sieiro P, Montalbán-López M, Mu D, Kuipers OP 2016 Bacteriocins of lactic acid bacteria: extending the family. Appl Microbiol Biotechnol 100:2939–2951. doi:10.1007/s00253-016-7343-9. 26860942 PMC4786598

[22] Ingram LC 1969 Synthesis of the antibiotic nisin: formation of lanthionine and beta-methyl-lanthionine. Biochim Biophys Acta 184:216–219. doi:10.1016/0304-4165(69)90121-4. 5791112

[23] Ingram L 1970 A ribosomal mechanism for synthesis of peptides related to nisin. Biochim Biophys Acta 224:263–265. doi:10.1016/0005-2787(70)90642-8. 5490258

[24] van der Donk WA, Nair SK 2014 Structure and mechanism of lanthipeptide biosynthetic enzymes. Curr Opin Struct Biol 29:58–66. doi:10.1016/j.sbi.2014.09.006. 25460269 PMC4267917

[25] Bauer R, Dicks LM 2005 Mode of action of lipid II-targeting lantibiotics. Int J Food Microbiol 101:201–216. doi:10.1016/j.ijfoodmicro.2004.11.007. 15862882

[26] Egan K, Ross RP, Hill C 2017 Bacteriocins: antibiotics in the age of the microbiome. Emerg Top Life Sci 1:55–63. doi:10.1042/ETLS20160015. 33525813

[27] O'Connor PM, O'Shea EF, Guinane CM, O'Sullivan O, Cotter PD, Ross RP, Hill C 2015 Nisin H is a new nisin variant produced by the gut-derived strain Streptococcus hyointestinalis DPC6484. Appl Environ Microbiol 81:3953–3960. doi:10.1128/AEM.00212-15. 25841003 PMC4524162

[28] Nishida H 2012 Comparative analyses of base compositions, DNA sizes, and dinucleotide frequency profiles in archaeal and bacterial chromosomes and plasmids. Int J Evol Biol 2012:342482. doi:10.1155/2012/342482. 22536540 PMC3321278

[29] Kuipers OP, Rollema HS, Yap WM, Boot HJ, Siezen RJ, de Vos WM 1992 Engineering dehydrated amino acid residues in the antimicrobial peptide nisin. J Biol Chem 267:24340–24346. 1447185

[30] Li Q, Montalban-Lopez M, Kuipers OP 2018 Increasing the antimicrobial activity of nisin-based lantibiotics against Gram-negative pathogens. Appl Environ Microbiol 84:e00052-18. doi:10.1128/AEM.00052-18. 29625984 PMC5981070

[31] Stein T, Heinzmann S, Düsterhus S, Borchert S, Entian K-D 2005 Expression and functional analysis of the subtilin immunity genes *spaIFEG* in the subtilin-sensitive host *Bacillus subtilis* MO1099. J Bacteriol 187:822–828. doi:10.1128/JB.187.3.822-828.2005. 15659659 PMC545732

[32] Otto M, Peschel A, Götz F 1998 Producer self-protection against the lantibiotic epidermin by the ABC transporter EpiFEG of Staphylococcus epidermidis Tü3298. FEMS Microbiol Lett 166:203–211. doi:10.1111/j.1574-6968.1998.tb13891.x. 9770275

[33] Stein T, Heinzmann S, Solovieva I, Entian KD 2003 Function of Lactococcus lactis nisin immunity genes nisI and nisFEG after coordinated expression in the surrogate host Bacillus subtilis. J Biol Chem 278:89–94. doi:10.1074/jbc.M207237200. 12379654

[34] Siegers K, Entian KD 1995 Genes involved in immunity to the lantibiotic nisin produced by Lactococcus lactis 6F3. Appl Environ Microbiol 61:1082–1089. 7793910 10.1128/aem.61.3.1082-1089.1995 PMC167363

[35] Ebner P, Reichert S, Luqman A, Krismer B, Popella P, Götz F 2018 Lantibiotic production is a burden for the producing staphylococci. Sci Rep 8:7471. doi:10.1038/s41598-018-25935-2. 29749386 PMC5945643

[36] Wysocki VH, Resing KA, Zhang Q, Cheng G 2005 Mass spectrometry of peptides and proteins. Methods 35:211–222. doi:10.1016/j.ymeth.2004.08.013. 15722218

[37] Field D, Blake T, Mathur H, O'Connor PM, Cotter PD, Ross RP, Hill C 2018 Bioengineering nisin to overcome the nisin resistance protein. Mol Microbiol 111:717–731. doi:10.1111/mmi.14183. 30537404

[38] Simões PM, Lemriss H, Dumont Y, Lemriss S, Rasigade J-P, Assant-Trouillet S, Ibrahimi A, El Kabbaj S, Butin M, Laurent F 2016 Single-molecule sequencing (PacBio) of the Staphylococcus capitis NRCS—a clone reveals the basis of multidrug resistance and adaptation to the neonatal intensive care unit environment. Front Microbiol 7:1991. doi:10.3389/fmicb.2016.01991. 28018320 PMC5157051

[39] Rink R, Wierenga J, Kuipers A, Kluskens LD, Driessen AJM, Kuipers OP, Moll GN 2007 Dissection and modulation of the four distinct activities of nisin by mutagenesis of rings A and B and by C-terminal truncation. Appl Environ Microbiol 73:5809–5816. doi:10.1128/AEM.01104-07. 17660303 PMC2074915

[40] Field D, 'O'Connor PM, Cotter PD, Hill C, Ross RP 2008 The generation of nisin variants with enhanced activity against specific Gram-positive pathogens. Mol Microbiol 69:218–230. doi:10.1111/j.1365-2958.2008.06279.x. 18485077

[41] Parente E, Hill C 1992 A comparison of factors affecting the production of two bacteriocins from lactic acid bacteria. J Appl Bacteriol 73:290–298. doi:10.1111/j.1365-2672.1992.tb04980.x.

[42] Field D, Begley M, O’Connor PM, Daly KM, Hugenholtz F, Cotter PD, Hill C, Ross RP 2012 Bioengineered nisin A derivatives with enhanced activity against both Gram positive and Gram negative pathogens. PLoS One 7:e46884. doi:10.1371/journal.pone.0046884. 23056510 PMC3466204

[43] Ryan MP, Rea MC, Hill C, Ross RP 1996 An application in cheddar cheese manufacture for a strain of Lactococcus lactis producing a novel broad-spectrum bacteriocin, lacticin 3147. Appl Environ Microbiol 62:612–619. 8593062 10.1128/aem.62.2.612-619.1996 PMC167827

[44] Lugli GA, Milani C, Mancabelli L, van Sinderen D, Ventura M 2016 MEGAnnotator: a user-friendly pipeline for microbial genomes assembly and annotation. FEMS Microbiol Lett 363:fnw049. doi:10.1093/femsle/fnw049. 26936607

[45] Lowe TM, Eddy SR 1997 tRNAscan-SE: a program for improved detection of transfer RNA genes in genomic sequence. Nucleic Acids Res 25:955–964. doi:10.1093/nar/25.5.955. 9023104 PMC146525

[46] Besemer J, Borodovsky M 1999 Heuristic approach to deriving models for gene finding. Nucleic Acids Res 27:3911–3920. doi:10.1093/nar/27.19.3911. 10481031 PMC148655

[47] Altschul SF, Madden TL, Schaffer AA, Zhang J, Zhang Z, Miller W, Lipman DJ 1997 Gapped BLAST and PSI-BLAST: a new generation of protein database search programs. Nucleic Acids Res 25:3389–3402. doi:10.1093/nar/25.17.3389. 9254694 PMC146917

[48] Mulders JWM, Boerrighter IJ, Rollema HS, Siezen RJ, de Vos WM 1991 Identification and characterization of the lantibiotic nisin Z, a natural nisin variant. Eur J Biochem 201:581–584. 1935953 10.1111/j.1432-1033.1991.tb16317.x

[49] De Kwaadsteniet M, Ten Doeschate K, Dicks LMT 2008 Characterization of the structural gene encoding nisin F, a new lantibiotic produced by a Lactococcus lactis subsp. lactis isolate from freshwater catfish (Clarias gariepinus). Appl Environ Microbiol 74:547–549. doi:10.1128/AEM.01862-07. 18039827 PMC2223265

[50] Zendo T, Fukao M, Ueda K, Higuchi T, Nakayama J, Sonomoto K 2003 Identification of the lantibiotic nisin Q, a new natural nisin variant produced by Lactococcus lactis 61-14 isolated from a river in Japan. Biosci Biotechnol Biochem 67:1616–1619. doi:10.1271/bbb.67.1616. 12913315

[51] Wirawan RE, Klesse NA, Jack RW, Tagg JR 2006 Molecular and genetic characterization of a novel nisin variant produced by Streptococcus uberis. Appl Environ Microbiol 72:1148–1156. doi:10.1128/AEM.72.2.1148-1156.2006. 16461661 PMC1392965

[52] Wu Z, Wang W, Tang M, Shao J, Dai C, Zhang W, Fan H, Yao H, Zong J, Chen D, Wang J, Lu C 2014 Comparative genomic analysis shows that Streptococcus suis meningitis isolate SC070731 contains a unique 105 K genomic island. Gene 535:156–164. doi:10.1016/j.gene.2013.11.044. 24316490

[53] Zhang Q, Yu Y, Velasquez JE, van der Donk WA 2012 Evolution of lanthipeptide synthetases. Proc Natl Acad Sci 109:18361–18366. doi:10.1073/pnas.1210393109. 23071302 PMC3494888

[54] Hatziioanou D, Gherghisan-Filip C, Saalbach G, Horn N, Wegmann U, Duncan SH, Flint HJ, Mayer MJ, Narbad A 2017 Discovery of a novel lantibiotic nisin O from Blautia obeum A2-162, isolated from the human gastrointestinal tract. Microbiology 163:1292–1305. doi:10.1099/mic.0.000515. 28857034 PMC5882112

